# Prolonged field care for traumatic extremity injuries: defining a role for biologically focused technologies

**DOI:** 10.1038/s41536-020-00117-9

**Published:** 2021-02-04

**Authors:** Connor P. Dolan, Michael S. Valerio, W. Lee Childers, Stephen M. Goldman, Christopher L. Dearth

**Affiliations:** 1 DoD-VA Extremity Trauma and Amputation Center of Excellence, Bethesda, MD USA; 2 Department of Surgery, Uniformed Services University of the Health Sciences and Walter Reed National Military Medical Center, Bethesda, MD USA; 3 Center for the Intrepid, Brooke Army Medical Center, San Antonio, TX USA

**Keywords:** Regenerative medicine, Translational research

## Abstract

Extremity injuries occur frequently during warfare. While traditionally treated in definitive clinics, the future battlefield is projected to be different in a variety of ways, and there will likely be a shift towards prolonged field care (PFC) for treating extremity traumas. PFC is defined as field medical care that is applied beyond “doctrinal planning time-lines” by a tactical medical practitioner in order to decrease patient mortality and morbidity. At present, there is an urgent need to develop biologically focused technologies for treating extremity injuries in the PFC setting. Herein, the case is made for why rapid advancements in PFC is critical to achieve optimal care for injured Service members in the future, and important design criteria for developing next-generation biologically focused technologies. Finally, a case example—i.e., Gustilo Type III fracture—is presented to illustrate how these biologically focused technologies could be utilized to treat an extremity injury within a PFC environment.

## Introduction

Mortality rates resulting from traumatic extremity injuries have decreased dramatically during operations: enduring freedom, Iraqi freedom, new dawn (OEF, OIF, and OND, respectively) relative to other conflicts from recent history (e.g., World War II, Vietnam War)^1^. This decrease is due, in part, to the 2009 announcement by then Secretary of Defense Robert Gates on the “Golden Hour” policy, which mandated that all injured US military personnel were to be evacuated from theater, the area where an armed conflict takes place, within 60 min of the time of injury^2^. In the years that followed this shift in policy, the average time to care was reduced by ~45 min and hundreds of lives were saved^3,4^.

Projections suggest, however, that the characteristics of a future, multi-domain battlefield against a peer or near-peer adversary (e.g., a country with comparable military power) may be quite different from that of OEF/OIF/OND^5^. Namely, many of the operational constructs that made “Golden Hour” evacuation possible such as air superiority, relatively small theater, and forward surgical units will not be guaranteed within future conflicts. Specifically, future conflicts are likely to bring about several new operational challenges. First, ground operations are likely to be ongoing across several fronts, and the theater will likely be larger than what was encountered during recent conflicts^6^. Second, near-peer adversaries are likely to rival the sophistication of our air power making reliable aeromedical evacuation scarce. Third, the added threat of cyberwarfare will make communication and rescue missions more challenging. Finally, the scale of attacks may prove more devastating than recent conflicts resulting in an astounding number of casualties that could challenge the bandwidth the US medical corps to provide the highest level of individualized care to injured service members (SM)^7^. Collectively, this means that evacuation of an injured SM will be, at best, challenging, if not impossible. As a result, it may be required that treatment of injured SM either be delayed or carried out on the battlefield for an extended period of time—a concept coined as prolonged field care (PFC). More specifically, PFC is defined as field medical care that is applied beyond “doctrinal planning time-lines” by tactical medical practitioner (TMP; e.g., Army Combat Medic (68 W), Special Operation Combat Medic (SOCOM/18D), Air Force Pararescuemen (PJs), Navy Corpsmen and Special Amphibious Reconnaissance Corpsman (SARC), etc) in order to decrease patient mortality and morbidity^6^. The ability to perform PFC successfully is confounded by a number of factors, including a lack of resources (i.e., limited to contents of SM’s rucksack) and an austere operational environment that is inherently non-sterile and likely to promote infection and acute complications (e.g., hyperthermia, hypothermia, dehydration). This scenario is further complicated by a lack of air superiority, in that units must remain mobile to minimize further attacks and additional unit casualty burden. As such, the prospect of a combat casualty needing to be cared for in such an environment for an extended period of time is suboptimal, as minimizing the time to surgery is a known correlate of surgical complications^8^.

Thus, the challenge currently facing the medical and research communities is how to maintain historic Golden Hour survivability rates within a resource limited PFC environment. Accordingly, much of the research investment to date within this domain has been focused on aspects of PFC that are directly associated with survivability (e.g., hemorrhage control and resuscitation). Beyond survivability, however, additional high priority requirements exist, which are in need of innovative solutions. Specifically, knowledge and/or materiel solutions are desperately needed which can (1) facilitate accelerated healing or off-loading of traumatic musculoskeletal wounds so as to allow injured SM to return to duty more expeditiously and (2) reduce the burden of survivorship by improving the quality of outcomes. Of note, given the likely nature of PFC within future battlefields, such solutions should be able to be applied wherever the casualties may occur without focusing on traditionally fixed echelons of care^7^. Clearly, numerous technical and operational challenges exist to be able to accomplish these goals, and thus it is clear that a wide-ranging portfolio of innovative solutions are in need of development in order to facilitate mission success. Of the many needed innovations, development, validation, and commercialization of a suite of biologically focused technologies tailored to traumatic musculoskeletal injuries within a PFC scenario are of the upmost need. These rationally designed biologically focused technologies will need to facilitate both: (1) enhanced short-term functionality of injured SM, which will have direct beneficial impacts on the readiness and lethality of the Joint force with respect to return to duty and return to combat rates; and (2) maximized long-term outcomes, including function and quality of life, following traumatic extremity injuries. Herein, key design criteria for PFC-specific biologically focused technologies will be discussed. Subsequently, and for illustrative purposes, a case example of how these design criteria might be applied to challenges associated with the management of a Gustilo Type III fracture to the extremity, a hallmark of combat-related musculoskeletal trauma, will also be discussed.

## Design criteria for biologically focused technologies of the future battlefield

Under doctrinal planning guidelines, combat casualty care of the most severe musculoskeletal injuries would occur within the confines of a forward surgical unit (Level II + ) within hours of wounding. Traditional forward surgical units are comprises of a 20-person multi-disciplinary team consisting of surgeons, nurses, surgical technicians, medics, and administrative officers, and are typically well stocked with all of the necessary medical equipment to perform damage control surgery in a sterile manner. In contrast, within a PFC scenario, a single TMP, who has limited access to supplies and resources, will likely be the one to provide care for an injured SM. As such, the current extent of medical interventions that could plausibly be provided to a SM with a traumatic extremity injury would be limited to procedures such as splinting, wound dressing, and perhaps wound debridement and/or fasciotomies (under the advisement of an orthopedic surgeon via telecommunication; if intact). To complicate matters further, all extremity wounds within a PFC environment would need to be assumed infected and, as such, primary closure cannot be performed. As such, the PFC scenario is not permissive traditional definitive musculoskeletal interventions such as autografting, orthobiologics, and cell-based therapies.

Given that traditional technologies and approaches for musculoskeletal trauma are not suitable for a PFC environment, a new class of PFC-specific technologies are in need of development. To enable accelerated return to duty of injured SM, these technologies need to focus on stabilization of the zone of injury from the perspective of controlling infection, retarding aberrant wound healing progression, preventing degeneration and/or atrophy of end organ function, and priming of the wound bed for positive surgical outcomes. However, given the resource and operational limitations associated with the PFC environment, traditional resource intensive approaches are not viable. Rather, this new class of biologically focused therapies need to be rationally designed to meet the needs of the end user (i.e., TMP and/or SM) and with the PFC operational environment in mind. To do so, several important design criteria need to be considered (Table 1; criteria are listed in descending order with respect to their relative priority/importance). Specifically, materiel solutions must: (1) be self-administrable or within the capabilities of a TMP, (2) be long-term (i.e., weeks to months) shelf-stable across temperature extremes, (3) be light weight and low cube so as to be carried in a standard issue rucksack, (4) be able to be applied quickly, (5) be designed for universal dosing and/or delivery, where possible, and (6) require little to no electrical power. Stated differently, overly complicated and/or resource intensive biologically focused therapies, even if effective in a controlled, fixed facility (i.e., Role 5) setting, are likely to be of little, if any, value to the PFC scenario. Additionally, when at all possible, therapies should be designed to facilitate a rapid restoration of function (e.g., mobility) to the affected extremity. In practice, this could either mean directly contributing to the restoration of function or indirectly contributing to limb function by not interfering with or being diminished by limb stabilization and/or off-loading technologies. The additional challenges posed by these criteria significantly increases the difficulty of designing effective treatments for injuries that are inherently difficult to treat in an ideal environment, however, achieving a solution to such design criteria is ultimately critical for optimizing musculoskeletal outcomes within a PFC setting.

**Table 1:** Design criteria for biologically focused technologies to be developed for use in PFC.

Design criteria	Rationale	Suggested characteristics
Self-administrable or within capabilities of a combat-medic	If SM’s are unable to receive medical attention, or if they are not able to reach a higher level of care, they will need technologies that they can use independently	Injectable, aerosol, powder, topical, etc.
Shelf-stable across a range of temperatures	Future theaters are uncertain, and thus technologies should be designed for a vast range of temperatures and climates	−30 to 50 °C
Light weight and low cube size	Reduce weight burden in SM’s rucksack	<1 kg
Rapidly administrable	To begin treating the injury as quickly as possible	Seconds– minutes
Universal dosing	For both ease of use and for making universal therapies	Effective dose«maximum tolerable dose
Zero or minimal electrical power	Access to reliable power is not guaranteed	No external power source required
Restore function of injured limb	To allow SM’s to safely evacuate dangerous areas and/or return to battle	Ability to independently ambulate

## Early interventions for gustilo type III fractures: a case example for implementation of PFC-specific design criteria for biologically focused technologies

In the early years of the recent conflicts, there were 758 reported cases of open fractures, 48% of which occurred in the tibia and fibula^9^. As such, these injuries represent a classic battlefield injury that is both highly prevalent and useful as a construct for a case example of how biologically focused interventions would be implemented within a PFC environment.

Battlefield open tibia fractures have the highest rate of infection (20–30%), and often lead to poor healing outcomes and delayed amputation^10,11^. Within the PFC environment, infection mitigation will be paramount as unresolved musculoskeletal infections are a leading cause of fracture non-union^12^ and deficient wound healing within the soft tissue compartment^13,14^.

Within a traditional forward surgical unit, early treatment of these injuries involves irrigation, wound debridement, and infection management followed by skeletal stabilization for transport^15^. In a PFC setting, these same principles will be critical for the efficacy of any proposed early interventions, with early fracture stabilization and infection control being of particular importance. While the regenerative medicine community has developed an armamentarium of orthobiologic and cell-based therapies to address type III open fractures, the development of such intervention has rarely considered the confounding impact of an active infection within the wound bed. Therefore, any proposed therapeutic should likely include an antibiotic/infection management component, or at a minimum, should not be diminished by concurrent infection management.

Definitive fixation is highly unlikely to be performed in the PFC setting and thus current fracture stabilization options are limited to splinting, casting, and/or limited external fixation^15–18^. These current treatment options typically require the injured SM to remain on a litter and thus are insufficient in future multi-domain battlefields where unit independence and mobility will be a necessity^5^. This creates an opportunity to develop field improvised or modular exoskeletal systems that can mechanically off-load injured tissues and enable SM mobility. In practice, these modular exoskeleton systems would be available to, and handled by, combat units similar to currently available collapsible litters systems (e.g., TALON II Model 90C, North American Rescue LLC, Greer, SC, USA) in that they would be carried by unit members during dismounted patrols or kept in a nearby vehicle^19^. There is also the possibility that these wound stabilizing and/or mobility enabling exoskeleton systems could be fabricated on-site by members of the combat unit by repurposing materials available to them on the battlefield. Therefore, similar to working in parallel with antibiotics/infection management, biologically focused therapies for PFC should work cooperatively with temporary stabilization and/or off-loading devices.

Interventions applied during this time period should also be focused on mitigating pathological and/or degenerative processes and promoting a pro-healing state. The opportunities for innovation in this space is immense, including, but not limited to, the development of: (1) a range of smart biomaterials with applications ranging from injury site stabilization to controlled release platforms for concomitant and/or temporally sequence delivery of bioactive payloads, and (2) shelf-stable small molecules and peptides capable of being delivered in such a manner that serve to modulate the local immune-inflammatory environment, mitigate fibrosis, prevent degeneration of neuromuscular junctions and/or modulate of the cell fate processes of progenitor cell populations between quiescence and activation/proliferation, as desired. It should be noted, of course, that for some of these PFC-specific requirements, it is clear that new technologies need to be developed de novo given the unique and novel nature of the needed capabilities; whereas for others, it is plausible that existing technologies (e.g., fracture putty, muscle void fillers, nerve glue, scaffold materials, etc) may be suitable, either in their current form or perhaps with slight modifications. Regardless of the biotechnologies investigators choose to develop and/or retrofit to address this unmet need, it is critical that they remember that therapies should be developed following the previously defined general design criteria (i.e., shelf-stable, low cube, self-administrable, etc.).

For some casualties, fracture stabilization, infection management, and administration of therapies for modulating the microenvironment of the zone of injury will represent the extent to which the injuries can be managed in a PFC Environment. For others, however, accelerated restoration of independent mobility represents an additional opportunity for innovation. While rapid and full return of mobility is desired, at a minimum, interventions that promote sufficient restoration of mobility to allow wounded SM to seek safe harbor and/or defend themselves from adversaries would be incredibly useful. For injuries affecting the lower extremities, this means that either the therapeutic intervention itself should either (1) impart functional capacity (e.g., load bearing, nerve conduction, generation of contractile force) via its intrinsic properties or (2) mediate rapid restoration of endogenous function in concert with a field-able off-loading devices (e.g., exoskeletons) capable of supporting tactical mechanical loading.

One analogy for conceptualizing the innovations that are necessary in this space is the Masquelet technique. The Masquelet technique is a staged surgical approach that relies on the placement of a cement spacer within the defect region of segmental fractures to prevent fibrous filling of the gap and induce a foreign body reaction so that a vascularized membrane is formed, increasing the efficacy of subsequent bone grafting at a later date. It is important to note that the cement spacer is temporary in nature and not specifically designed to be osteoconductive, osteoinductive, or osteogenic in the sense that classical definitive treatment might be. Initially the composition of such cements was relatively inert as the intent was structural in nature. However, as the approach grew in popularity a number of investigators have begun to modify the spacer material and topography so as to alter cell adhesion, which in turn affects the growth factor and basal protein composition of the resulting vascularized membrane. Moreover, spacers capable of antibiotic release to combat deep, persistent infections have also been tested. Furthermore, nothing about such an approach would inherently limit the ability of the wounded SM to simultaneously attach an off-loading device (e.g., exoskeletons) that might enable immediate mobility provided if sufficient fracture stabilization and pain management is achieved. Thus, while the Masquelet technique is not presently suited to be performed safely in a PFC setting, the biomaterial spacers used therein exhibit several key design concepts that the scientific community are well equipped to adapt and apply in novel ways so as to develop new technologies that can be applied in a PFC scenario.

## Conclusions

Future battlefields will be associated with an increase in time to evacuation of injured SMs, and thus PFC will be prevalent. As it stands today, current technologies and approaches are insufficient to provide adequate medical care within a PFC environment and the capability gap between definitive treatments available in a fixed facility (i.e., Role 4) environment versus short-term treatment in the PFC environment is too wide. Therefore, an urgent need exists for rapid improvements in biologically focused technologies that can be effectively used in an austere PFC environment. In designing biotechnologies, investigators should design their devices to be self- and rapidly administrable, shelf-stable, light weight, universal dosing, requires minimal power, and if possible, restore limb function. Significant time, effort, innovation, and financial resources need to be invested towards developing and commercializing these next-generation technologies. In doing so, clinicians and investigators must be cognizant of the challenges associated with PFC, and thus design biologically focused therapeutics for a broad range of tissue injuries with these design criteria in mind.

## References

[1] Devore DI (2011). For combat wounded: extremity trauma therapies from the USAISR. Mil. Med..

[2] Gates, R. M. *Duty: Memoirs of a Secretary at War*. 304–305 (Random House, 2014).

[3] Kotwal RS (2016). The effect of a golden hour policy on the morbidity and mortality of combat casualties. JAMA Surg..

[4] Howard JT (2019). Use of combat casualty care data to assess the US military trauma system during the Afghanistan and Iraq conflicts, 2001-2017. JAMA Surg..

[5] US Department of the Army, Training and DoctrineCommand (TRADOC) Pamphlet 525–3–1: The US Army in Multi-Domain Operations 2028 (Fort Eustis, VA: Government Printing Office, 2018). https://www.tradoc.army.mil/portals/14/documents/mdo/tp525-3-1_30nov2018.pdf.

[6] Keenan S, Riesberg JC (2017). Prolonged field care: beyond the “golden hour”. Wilderness Environ. Med..

[7] Rasmussen TE, Baer DG, Cap AP, Lein BC (2015). Ahead of the curve: Sustained innovation for future combat casualty care. J. Trauma Acute Care Surg..

[8] D’Alleyrand JC (2014). Is time to flap coverage of open tibial fractures an independent predictor of flap-related complications?. J. Orthop. Trauma.

[9] Owens BD, Kragh JF, Macaitis J, Svoboda SJ, Wenke JC (2007). Characterization of extremity wounds in operation Iraqi freedom and operation enduring freedom. J. Orthop. Trauma.

[10] Patzakis MJ, Wilkins J (1989). Factors influencing infection rate in open fracture wounds. Clin. Orthop. Relat. Res.

[11] Burns TC (2012). Microbiology and injury characteristics in severe open tibia fractures from combat. J. Trauma Acute Care Surg..

[12] Giannoudis PV, Papakostidis C, Roberts C (2006). A review of the management of open fractures of the tibia and femur. J. Bone Jt. Surg. Br. Vol..

[13] Jin RM, Warunek J, Wohlfert EA (2018). Chronic infection stunts macrophage heterogeneity and disrupts immune-mediated myogenesis. JCI Insight.

[14] Zúñiga-Pereira AM, Santamaría C, Gutierrez JM, Alape-Girón A, Flores-Díaz M (2019). Deficient skeletal muscle regeneration after injury induced by a clostridium perfringens strain associated with gas gangrene. Infect. Immun..

[15] Osier C (2018). Orthopedic trauma: extremity fractures. Mil. Med..

[16] Possley DR (2010). Temporary external fixation is safe in a combat environment. J. Trauma.

[17] US Army. Survival, FM 3-05.70 (FM 21-76) (John F. Kennedy Special Warfare Center and School, 2002).

[18] US Army. Treating Fractures in the Field, MD0533 (U.S. Army Medical Department Center and School, 2008).

[19] Headquarters Department of the Army. CasualtyEvacuation (ATP 4–25.13) Army Techniques Publication No. 4–25.13, Feb 9, 2013. https://armypubs.army.mil/epubs/DR_pubs/DR_a/pdf/web/atp4_25x13.pdf.

